# World Society of Emergency Surgery-indication of globalism and renaissance through the 2015 biennial assembly

**DOI:** 10.1186/s13017-015-0035-4

**Published:** 2015-09-04

**Authors:** Yoram Kluger, Fausto Catena, Jeffry Kashuk, Luca Ansaloni

**Affiliations:** Rambam Health Care Center, POB 9602, Haifa, 31096 Israel; Parma University Hospital, Parma, Italy; Assia Medical Group, Assuta Medical Centers, Tel Aviv, Israel; Papa Giovanni XXIII Hospital, Bergamo, Italy

The World Society of Emergency Surgery (WSES) was established in 2007. The objectives set forth by the society, as enumerated in the mission statement, is: “The overall goals include the promotion of the specialty of emergency surgery as part of the emerging discipline of acute care surgery via academic exchange in an effort to further training and education as well as translational research in the specialty’.

The first WSES international assembly took place in Sardinia, Italy in 2011 and the second in Bergamo, Italy, in 2013.

The 3^rd^ international congress of WSES was held in Jerusalem, Israel, 5–8 July, 2015. As this was the first meeting outside of Italy, it represented an important worldwide extension of the society, with over 23 countries being represented by over 150 attendees. A pre- congress educational course on IAI (intra-abdominal infections) preceded the academic gathering, attended by 20 residents in training who enthusiastically participated and successfully completed the course. The residents were provided with a certificate of attendance by the WSES IAI course directors.

An important and carefully planned objective of the meeting involved presentation of evidence based guidelines for common clinical challenges such as acute appendicitis, acute cholecystitis, and acute diverticulitis. These guidelines were reached after a detailed and methodical process with involvement of many contributing physicians and institutions and summaries of the suggested guidelines were presented for the approval of the assembly. The resultant guidelines from this process will be submitted for publication in the forthcoming issues of the World Journal of Emergency Surgery.

Systematic reviews of the literature were presented in plenary sessions on a multitude of topics covering the entire spectrum of Emergency Surgery,

A two and half day academic meeting followed the course and included 35 position lectures and 8 WSES guidelines reports.

Eighty one clinical and research based abstracts were submitted from worldwide academic institutions, and 68 were presented in parallel sessions. Seven outstanding abstracts were chosen by the scientific committee and were presented in a plenary session. The assembly than nominated the three best abstract (1^rd^ -3^rd^ places) using an electronic voting system. The first authors of these abstracts were respected with a certificate signed by the congress chairmen.

Besides discussions of the surgical approach to a many clinical challenges, state of the art strategies dealing with virtually all areas of surgical emergencies were comprehensively discussed. Thought-provoking case discussions presented during the meeting provided a great opportunity to share extensive knowledge and personal experiences from esteemed colleagues worldwide.

Being a young society, the excitement generated by the meeting in Jerusalem reflected the dedication of its members to the specialty and generated new interest by a broader group of young surgeons and trainees. While the 2015 WSES meeting was termed “The Last of the Mohicans? - the General Surgeon of 2015?”, the keen support of many young surgeons suggests that indeed there is much enthusiasm and hope for the future of the specialty of Emergency Surgery.

The executive board voted to host the next meeting, in 2017, in Brazil.

We look forward to seeing everyone there as the congress moves to the American continent for the first time.

Please join us at the new WSES web site (www.wses.org.uk) and in all the society activities (WSES registries, multi-center trails, courses and meetings). Your membership in the society is important.

